# A Scoping Assessment of Implemented Toxicokinetic Models of Per- and Polyfluoro-Alkyl Substances, with a Focus on One-Compartment Models

**DOI:** 10.3390/toxics11020163

**Published:** 2023-02-09

**Authors:** Alexander East, Daniel E. Dawson, Sydney Brady, Daniel A. Vallero, Rogelio Tornero-Velez

**Affiliations:** 1 U.S. Environmental Protection Agency, Office of Research and Development, Center for Computational Toxicology and Exposure, 109 T.W. Alexander Drive, Research Triangle Park, NC 27709, USA; 2 Oak Ridge Associated Universities, Oak Ridge, TN 37830, USA; 3 ToxStrategies LLC, 31B College Place, Asheville, NC 28801, USA

**Keywords:** PFAS, toxicokinetics, scoping assessment

## Abstract

Toxicokinetic (TK) models have been used for decades to estimate concentrations of per-and polyfluoroalkyl substances (PFAS) in serum. However, model complexity has varied across studies depending on the application and the state of the science. This scoping effort seeks to systematically map the current landscape of PFAS TK models by categorizing different trends and similarities across model type, PFAS, and use scenario. A literature review using Web of Science and SWIFT-Review was used to identify TK models used for PFAS. The assessment covered publications from 2005–2020. PFOA, the PFAS for which most models were designed, was included in 69 of the 92 papers, followed by PFOS with 60, PFHxS with 22, and PFNA with 15. Only 4 of the 92 papers did not include analysis of PFOA, PFOS, PFNA, or PFHxS. Within the corpus, 50 papers contained a one-compartment model, 17 two-compartment models were found, and 33 used physiologically based pharmacokinetic (PBTK) models. The scoping assessment suggests that scientific interest has centered around two chemicals—PFOA and PFOS—and most analyses use one-compartment models in human exposure scenarios.

## 1. Introduction

Poly- and perfluoroalkyl substances (PFAS) are a family of more than 4730 highly fluorinated aliphatic compounds manufactured for a variety of diverse applications [1]. The list continues to expand in part due to advancements in non-targeted screening analytical techniques which have enabled identification of many previously unknown substances in environmental samples and consumer products (OECD 2021). Since the 1950s, these compounds have been widely used in consumer products such as water-repellent clothing, stain-resistant carpets, non-stick cookware, and oil resistant paper food packaging and for industrial products such as firefighting foams to extinguish fuel fires [2,3,4]. The carbon–fluorine bond is the strongest bond in organic chemistry, thus providing chemical and thermal stability to the perfluoroalkyl moiety [5]. The properties which make PFAS suitable for high temperature applications and corrosive environments also make them resistant to environmental and metabolic degradation [3,6]. Consequently, a broad range of these substances have been detected in the environment, wildlife, and humans [7,8]. As such, many PFAS have been detected with high frequencies in human serum in populations in the U.S. [9,10], Germany [11,12], Sweden [13], and China [14].

Currently, much of the human health effect data available for PFAS are for a handful of chemicals, primarily legacy PFAS such as perfluorooctanoic acid (PFOA, DTXSID8031865) and perfluorooctane sulfonate (PFOS, DTXSID3031864). As of this writing, the most up to date list of PFAS with peer reviewed health information on the EPA CompTox Dashboard is the PFAS-Tox Database, containing 44 different chemicals, only 29 of which are unique (i.e., some are salts) (https://pfastoxdatabase.org/, accessed on 15 December 2022). Epidemiological studies suggest an association between these legacy PFAS chemicals and immune suppression, thyroid disease, high cholesterol, ulcerative colitis, kidney cancer, testicular cancer, breast cancer, hypertension during pregnancy, decreased weight at birth, and increased risk of miscarriage [15,16].

The regulatory community has specifically recognized long-chain perfluoroalkyl sulfonic acids and perfluoroalkyl carboxylic acids as contaminants of high concern because they have been shown to be more bioaccumulative than their short-chain analogues [1]. PFOS and PFOA are the long chain perfluoroalkyl acids most often reported in the scientific literature [5,17]. Current half-life estimates of PFOA for humans vary from 1.2 and 1.7 years for males and females [18], respectively, to 3.9 years [19]. For PFOS, current half-life estimates vary from 2.91 years [20] to 6.3 and 22 years [18] for females and males, respectively. A compilation of half-lives for these and other PFAS for multiple species is recently provided by Dawson et al. [21]. Some short chain perfluoroalkyl acids have short elimination half-lives in humans and thus lower potential for bioaccumulation; for example, perfluorobutanoic acid (PFBA, DTXSID4059916) is eliminated with an estimated half-life of 72 days [22] and perfluorobutane sulfonate (PFBS, DTXSID5030030) is eliminated with an estimated half-life ranging from 0.12 days to 26 days for females and 28 days for males [23]. Accordingly, potential for bioaccumulation has been proposed as a grouping strategy for PFAS [15]. Others argue that their persistence in the environment provides sufficient basis for regulating PFAS as a class [6,8]. The EPA has proposed two approaches for initially grouping PFAS, one based on toxicity and toxicokinetic data, and another based on removal and remediation strategies [24].

To understand the mechanisms by which PFOA, PFOS, and other PFAS accumulate in humans, their toxicokinetics have been studied in several experimental animals. However, in rats, mice, monkeys and other animals, their half-lives are on the order of hours to days whereas in humans their half-lives may extend to several years [25,26]. This indicates substantial differences in toxicokinetic behavior between humans and other species. For decades, the mechanism of sex-, species-, and chain-length dependent renal elimination of PFAS has been an important area of research [27,28]. It is recognized that organic anion transport proteins play a key role in renal tubular reabsorption of certain PFAS [27,29,30]. However, the parameterization of mechanistic models of PFAS toxicokinetics (e.g., physiologically based toxicokinetic models (PBTK)) remains a challenge due to the relative lack of experimental toxicokinetic data for many PFAS and the large number of species-, sex-, and age-specific differences that have been observed [21]. Use of parsimonious models (i.e., one-compartment) is common in the literature for both commonly studied PFAS [31] and data poor scenarios, for which data is not readily available for model parameterization [15]. Given the wide diversity of PFAS and differences in TK, it is helpful moving forward to inventory the developed approaches and the relative focus paid to particular PFAS and model structures.

Using a systematic evidence-mapping approach [32], we conduct a scoping assessment of published studies that characterize the use of TK models of PFAS across multiple species. Following a general breakdown of major classes of models (e.g., one-compartment, two-compartment, PBTK), we focus on describing the canonical development of one-compartment models for PFAS using bibliometric techniques. From this analysis, we identify and describe key methodological developments of characterizing PFAS TK. Finally, we extract key model features, and discuss their application in future modeling work.

## 2. Materials and Methods

### 2.1. Literature Search and Screening

Papers for the systematic mapping were collected using the Web of Science (WOS) search tool with a search string based on a systematic review protocol developed by DeLuca et al. [33] and provided in the Supplemental Figure S1. The search query was inclusive of all PFAS and pharmacokinetic model types from 2000–2019 in December 2019. The body of papers returned from this search (*n* = 692) were imported into an EndNote library, screened for duplicates, and then imported into the SWIFT Review tool (*n* = 688) [34]. Machine learning tools in SWIFT Review were used to reduce the corpus to a more manageable subset. SWIFT-Review applies a bag-of-words model to characterize a document’s features [34]. In the bag-of-words model, unique terms (e.g., ‘toxicokinetic’) are identified in the title, abstract, and MESH headings; these include individual words and 2- and 3-g terms (e.g., ‘toxicokinetic modeling’, ‘physiologically based pharmacokinetic’). Given a training set of manually identified “relevant” and “non-relevant” documents, SWIFT-Review applies a log-linear model using the frequencies of the terms to compute the conditional probability (i.e., score) that a document is relevant [34]. We employed initial scores, prior to training, to help create the training set. First, the corpus was divided into upper, middle, and lower thirds based on their scores, representing high, medium, and low relevance to keywords. From this, a training set of 69 papers (approximately 10% of the entire corpus) was compiled by randomly selecting 23 papers selected from each bin. This training set of studies underwent a manual abstract screening by 2 independent reviewers to determine if the study met inclusion criteria. Inclusion criteria in this stage of screening was only that the abstract indicated use of a TK model for PFAS within the study. Discrepancies between the two reviewers’ inclusion or exclusion decisions were settled through discussion to produce a final list of studies to be used in the training set. The list of included/excluded training papers was then used to inform SWIFT Review’s machine learning model to predict the probability of inclusion of papers in the rest of the corpus without the reviewers having to manually screen all abstracts. 

Based on the behavior of our training set, we estimate that by setting the required inclusion probability to 0.5 we captured about 95% of relevant papers (Figure S2). This reduced the corpus to a subset of 174 papers, which was a reduction of approximately 75% of the original number of studies. When using a machine learning classifier there is a classic trade-off between precision and recall depending on the required inclusion probability [35]. A better quality of papers (i.e., higher proportion of relevant papers) may be screened by increasing the required inclusion probability, but only at the expense of missing some relevant papers (lower recall). Thus, we chose to set the bar low (0.5) on the classifier and follow up with a second manual abstract review using the same inclusion criteria as above; i.e., the abstract indicted the inclusion of a TK model involving PFAS within the study. This further reduced the number of included studies to 86. 

**Figure 1:**
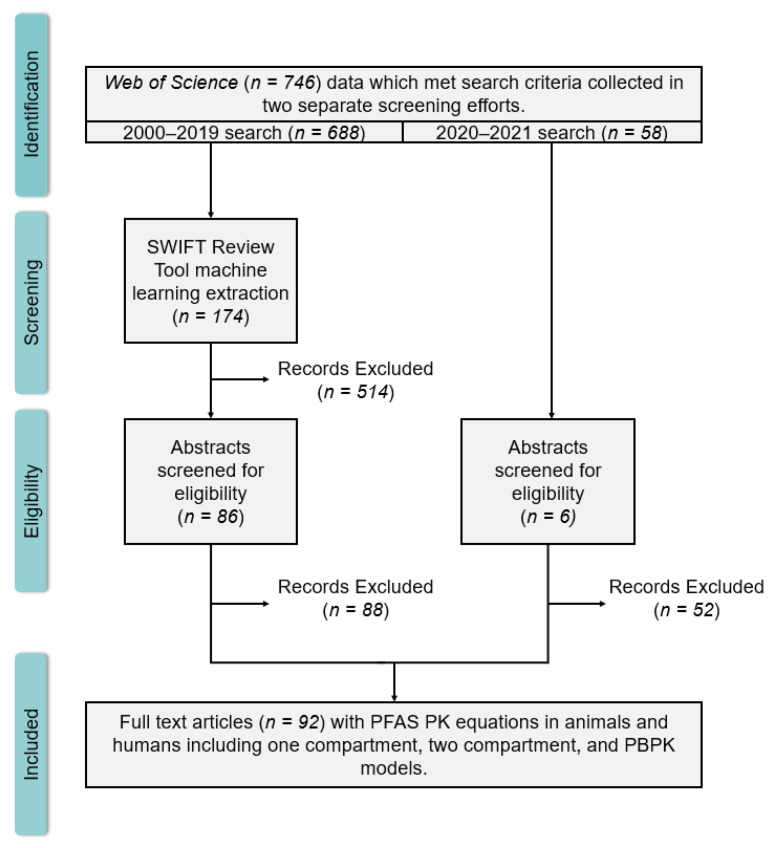
Identification, screening, eligibility, and inclusion of 2000–2019 and 2020–August 2021 PFAS TK WOS searches.

Finally, an additional literature search in WOS was conducted in July 2021 for studies published in the first half of 2020–2021 to identify any recently published studies that were not included in the previous literature search (*n* = 58). Due to the relatively small size of this additional search, it was subjected to only a manual abstract review and in the inclusion of 6 additional relevant papers. Combined, these search and screening activities resulted in a total corpus of 92 papers. The total literature search and screening effort is described in Figure 1. This corpus was then subjected to the full-text review and the model extraction described below.

### 2.2. Data Extraction and Curation

From each included paper at the full-text level, a suite of data was extracted and stored in an Excel spreadsheet. This data included: (1) basic bibliographic details such as title, authors, journal, publication year, location of publication, and number of references; (2) the type of model (one compartment, two compartment, or PBTK model), (3) the source of model (novel model or from a previously published source), (4) organism (animal or human), (5) the chemical(s) included, and (6) the analytical instrumentation used. Papers were subset by implementation of a one compartment model. Included papers develop new toxicokinetic models for PFAS, calibrate parameters, or leverage toxicokinetics to estimate dose or exposure (reverse dosimetry). 

Model type is determined by the number of compartments. A one compartment model will consider the body as a single ‘compartment’, whereas a two-compartment model typically consists of a central and peripheral compartment, or it depicts the relationship between two organisms (i.e mother-child). Both one and two compartment models employ the concept of volume of distribution (*V_D_*), a theoretical volume needed to contain the total mass of drug administered (or total intake of toxicant) at the concentration observed in the plasma. A value of *V_D_* comparable to the blood volume suggests that the drug/toxicant is confined to the blood, whereas a value comparable to the body volume indicates that the drug/toxicant distributes throughout the body. In contrast, PBTK models consider physiological volumes (e.g., kidney volume) explicitly and comprise more than two compartments. Additional information is available in the Supplemental Information (Table S2).

Equation (1) describes a simple one-compartment toxicokinetic model. This model was used by East et al. [36] to predict the concentration of a PFOA and PFOS in blood serum as a function of dose, elimination rate, and volume of distribution:

(1) $$\frac{d\left(C_{s}\right)}{dt}=\frac{D}{V_{D}}-k_{e}\times C_{s}$$

where *C_s_* is concentration in the serum (ng/mL), *D* is the daily intake dose (ng/kg-bw/day), *V_D_* is the volume of distribution (mL/kg), and *k_e_* is the elimination rate (1/day). At steady-state *d(Cs)/dt* = 0, giving the following:

(2) $$C_{s}=\frac{D}{k_{e}V_{D}}$$

In Equation (2), a lower value of *V_D_* would result in an increase in the steady state concentration, other parameters held constant. Since the drug/toxicant has less theoretical volume within which to distribute, it is more concentrated. Similarly, a lower elimination rate constant signifies less substance is eliminated over time, resulting in a higher steady state concentration. Equation (1) is specified with zero-order input *D* which is often practical for modeling human exposure scenarios. Alternatively, Equation (1) can also be specified with first order absorption to interpret animal studies by gavage as described by Wambaugh et al. [37]. Additionally, Wambaugh et al. [37] describe the differential equations for the central and peripheral concentrations of a two-compartment model which was used to describe the bi-phasic elimination of PFOA in male rats.

### 2.3. Analyses

Extracted data from the included studies were then analyzed using the systematic mapping methodology outlined in James et al. [32], which defines a ‘systematic map’ as a collation, description, and catalog of available evidence. This methodology shares similarities to a systematic review, with the primary difference being that it does not involve the evaluation of a hypothesis. A general comparison between a systematic review and a systematic mapping is provided in the Supplemental Information (Table S1). While the search strategy and article screening used here were adapted from DeLuca et al. [33], and were consistent with systematic review protocols, this entire effort most closely represents a systematic mapping.

A summary analysis regarding similarities in model structure, the prevalence of animal versus human studies, and frequency of the most common PFAS species studied are presented in the results. These descriptions of the corpus provide some description of the field. Model structures are classified as one compartment (OC), two compartment (TC), or physiologically based toxicokinetic (PBTK). Because some models leverage multiple model structures, flagging multiple structures occurs in this analysis. Two compartment models are inclusive of both physiological compartment models (kidney to liver) and transfers between unique receptors, such as mother to child. The most commonly modeled PFAS across publication year is also included. Again, because some manuscripts evaluate multiple PFAS, duplicate assignments are recorded. As such, the counts of model type and PFAS evaluated exceed the number of papers in the corpus. 

To analyze the bibliometric relationships between the authors of the included studies, the R Package *Bibliometrix* [38,39] and the corresponding RShiny application *Biblioshiny* [38] were used. Influential papers were identified using network analysis-based tools in *Bibliometrix*. This package allowed for the construction of networks of citations between papers in the corpus, with papers represented by nodes and citations between papers represented as edges between the papers. In this way, metrics such as centrality could be used to describe the relative influence that papers and/or TK models have had on the overall field of study. 

*Bibliometrix* network plots were used in two ways. First, after having gathered basic information on TK structures from the corpus, a co-citation network was leveraged to identify important papers outside the corpus. For this we examined the network of references co-cited by the corpus [40]. Articles not included in our corpus may have included data articles and review articles that provided a foundation for the TK models in the corpus but were not captured by the inclusion criteria. Additionally, missed implementations of TK models could potentially be identified that were inadvertently dis-included. The co-citation network was produced using the *Bibliometrix* function *networkPlot()*, with clustering set to “louvain”, and *n* = 20 nodes for interpretability [38]. The Louvain clustering algorithm [41] has been shown to be an optimal algorithm for community detection [42]. A ‘betweenness’ centrality score is produced for each node (publication), and nodes with high betweenness centrality serve as important connectors between other nodes [43,44]. In this case, such papers are commonly cited by other authors in the network, suggesting they are influential. Thus, articles may be identified which appear outside of the extracted corpus but which influenced PFAS TK modeling [45].

Secondly, given our focus on one compartment models for humans (OCH), we generated a visualization of publication activity over the years. This was accomplished with a ‘historiograph’, a chronological network map of the most relevant direct citations resulting from a bibliographic collection [38,46]. The visualization of OCH models was further augmented to show dynamical and steady state assignments with different colors and node size based on the ratio of local citations (LCS) to global citations (GCS). The LCS comprises citations within the corpus, and GCS comprises the total citations that an article in the corpus has received from documents indexed on a bibliographic database (Web of Science) [38]. Spacing between nodes was determined by Fruchterman–Reingold force direction within *Bibliometrix* [47].

In addition to the graphical summaries, manuscripts employing OCH models with over 50 global citations were pulled from *Bibliometrix* for further extraction to characterize the important applications of OCH models. The GSC, the basis for determining which models were selected, was recorded in the initial WOS search performed in December 2019, and several commonly cited publications have exceeded this threshold since the WOS search. The purpose of this subset is to evaluate OCH PFAS TK models that have greater influence on other research communities. 

## 3. Results

### 3.1. Corpus of Extracted Texts

Descriptive statistics on the corpus generated by the *Bibliometrix* package are shown in Table 1, The body of included publications from the WOS search and subsequent screening processes contained 92 articles with 358 unique authors, and a combined set of 2670 references which were mined for co-citation analysis. Of the 92 articles, there are 50 studies with one-compartment models, 17 with two compartment models, and 33 with PBTK models. Some articles contained more than one model structure and were double counted, so the total count of models exceeds the number of papers within the corpus.

**Figure 2:**
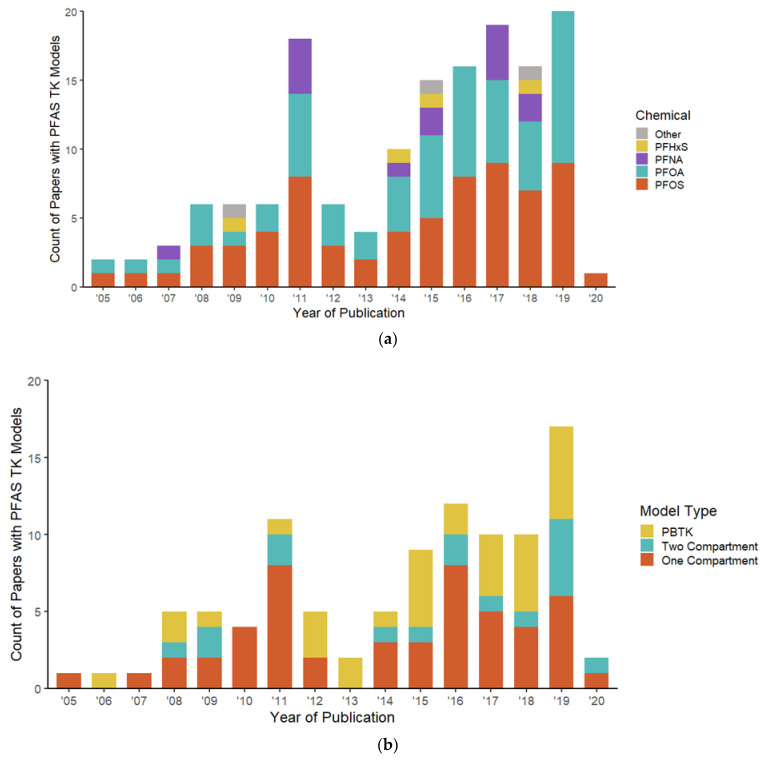
Descriptive statistics on the corpus for years 2005 through 2020: (**a**) Chemical and PFAS analyzed in pharmacokinetic models across publication year; (**b**) Model type and PFAS used in pharmacokinetic models across publication year.

The most common chemical modeled was PFOA, which was included in 69 of the 92 analyses, followed by PFOS being included in 60 analyses. Perfluorohexanesulfonic acid (PFHxS, DTXSID7040150) was reported in 22 manuscripts and perfluorononanoic acid (PFNA, DTXSID8031863)) was present in 15. Only 4 of the 92 papers did not include analysis of PFOA, PFOS, PFNA, or PFHxS (Figure 2a). Most publications with a PFAS TK model are from the U.S. (41), followed by Sweden (8), China (6), Germany (6), and Korea (5). Although the WOS searches spanned from 2000–2021, the earliest detected PFAS TK model was in 2005. Figure 2b shows model type across year for all included papers in the scoping assessment.

**Figure 3:**
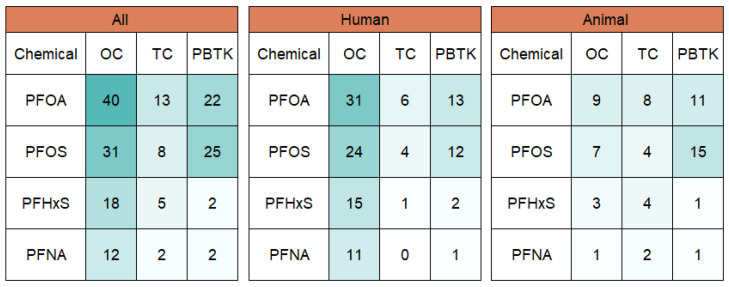
Chemical and TK model type by organism facet for the four most studied PFAS. Note: OC = One Compartment, TC = Two Compartment, PBTK = Physiologically Based Toxicokinetic.

In the included corpus, 55 of the 92 manuscripts recorded use of PFAS TK models for humans and 43 used models for animal studies. A breakdown of TK model types by organism and common PFAS chemicals is presented in Figure 3. One-compartment models using PFOA and PFOS were the most common across all organism models and human models. Animal studies were more likely to use PBTK models for both PFOA and PFOS. Animal species is available in the supplemental information. 

### 3.2. Exploring Evidence: Influential Publications Co-Citation

**Figure 4:**
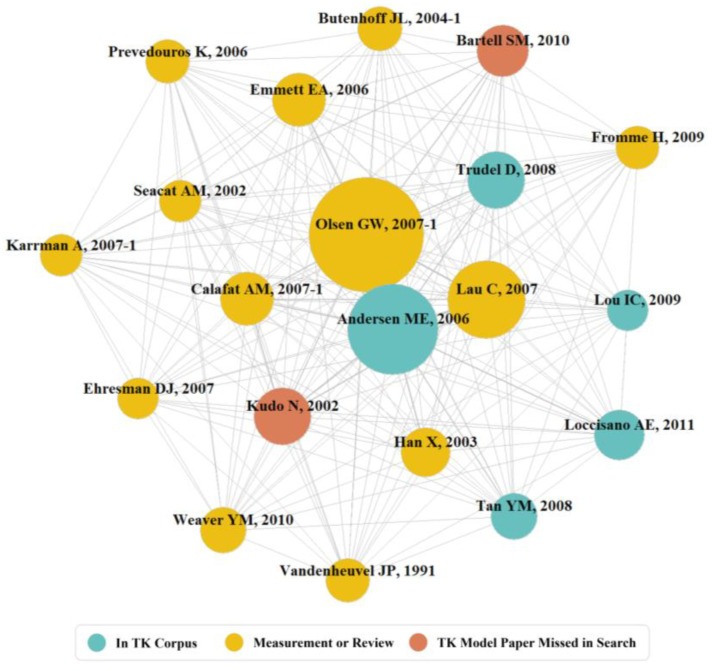
Co-citation network from entire corpus (top 20 nodes) [4,28,30,48,49,50,51,52,53,54,55,56,57,58,59,60,61,62,63,64].

The co-citation network in Figure 4 is color coded: blue publications (nodes) are within the extracted corpus of 92 papers. Yellow nodes are review or measurement publications outside of the extracted corpus. The two red nodes are implementations of TK PFAS models not included in the corpus. 

Measures of the network centrality of the 20 publications depicted in Figure 4 are provided in Table 2, The table is flagged with the same color scheme used in the co-citation network depicted in Figure 4. Manuscripts which comprise the PFAS TK modeling community are often data publications commonly used to bound exposure estimates [9,51,54,56] though some offer models and parameters for PBTK models [28,55,63,64]. Despite the high proportion of publications using one-compartment models, PBTK models feature strongly in the co-citation network. Trudel et al. [61] use a one-compartment model to estimate consumer exposure to PFOA and PFOS, the two most modeled PFAS. Despite being commonly cited by authors in the network, two articles employing TK for PFAS failed to be included in the corpus (92 articles). Both studies involve application rather than development of TK. Kudo et al. [28] make scarce mention of the two-compartment model used for determination of renal clearance of PFOA in rats. Bartell et al. [60] applied a mixed-effect model to estimate the decline in serum PFOA, and adjust for covariates, for individuals served by public water systems testing the use of granulated carbon from 2007–2008. 

None of the yellow or red node publications depicted in Figure 4 were retrieved in the WOS search, yet all are cataloged in WOS. In the case of yellow nodes this is good because we sought papers applying or developing TK models of PFAS, and thus these papers could be considered true negatives. The sole exception to this was Olsen et al. [56] which was retrieved in the WOS search, scored high in SWIFT-Review, but pulled in manual curation (see Table S4). The red node publications (Bartell et al. [60] and Kudo et al. [28]) were not retrieved from WOS even though they met our inclusion criteria; thus, they are false negatives. Papers identified as the 20 most central in the co-citation network are older papers (<2012) and therefore the most central nodes reflect the more seminal works in the field which have garnered citations over time. Discovering additional relevant papers in the co-citation network could be achieved by expanding the number of nodes examined.

### 3.3. A Chronology of One-Compartment Models

**Figure 5:**
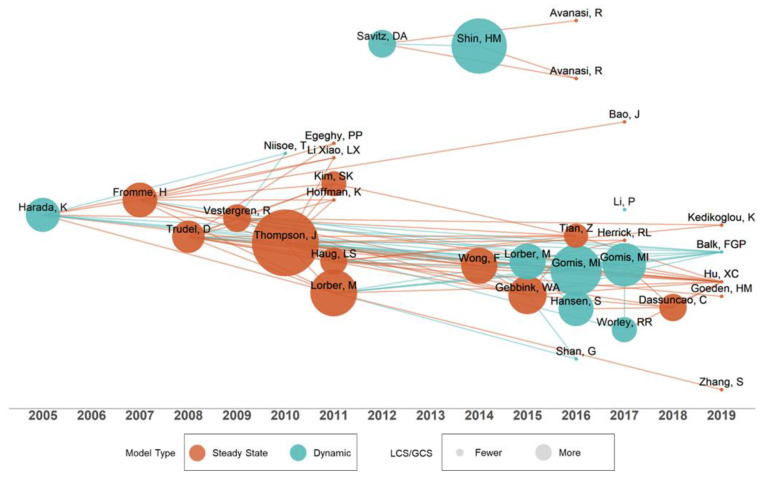
Historiograph of human one compartment TK models used for PFAS (2005–2019) [14,19,25,61,65,66,67,68,69,70,71,72,73,74,75,76,77,78,79,80,81,82,83,84,85,86,87,88,89,90,91,92,93,94].

The chronology and relationships between publications containing one-compartment models for humans are captured in the historiography depicted in Figure 5. Node size represents the count of LCS, or citations by other texts in the corpus, to the total number of citations GCS. The color represents steady state or dynamic models. Lines between publications signify a citation from the paper on the right. The line color depends on the origin of the line, showing the influence of model types. 

With an LCS-to-GCS ratio of 25%, Thompson et al. [67] was the largest node in Figure 5. The authors applied a one compartment approach to estimate total intakes of PFOA and PFOS for the general population of urban areas on the east coast of Australia. The study employed an elimination rate constant of 0.0008 day^−1^ for PFOA and 0.0003 day^−1^ for PFOS, and values of 170 and 230 mL/kg-bw for the volume of distribution, respectively. We note that a one-compartment TK model is fully specified with biological half-life (t12) and *V_D_*. Note that the elimination rate constant (k_e_) is inversely related to the biological half-life, t12 = *ln*(2)/*k_e_*. The isolated cluster at the top of the plot is a collection of papers on the C8 Health project, an epidemiological study with 69,030 individuals enrolled over a 13-month period from 2005–2006 [75]. We identified three other epidemiological studies employing ‘mother-child’ models by TC, PBTK or both (see Table S2). There has been an observable shift in the literature from steady state to dynamic models. In the period prior to 2012, there were 3 dynamic model publications and 10 steady state (23%). After 2012, there were 8 dynamic and 13 steady state (38%). Some models exist outside the corpus. For example, Harada et al. [25] reference the PFAS TK model used in Harada et al. [95], which was not captured by the scoping assessment. As such, the historiograph in Figure 5 is best interpreted as a simple chronology of influential publications which incorporate one-compartment human TK models for PFAS rather than a total diagram of the published literature. Furthermore, as lines between nodes are representative of manuscript citations, the links drawn are not inherently references of model use. 

Additional evaluation of influential publications of OCH PFAS TK was conducted on a subset of models with greater than 50 global citations (Table 3). These papers represent prevalent OCH PFAS TK publications among the fields of exposure, toxicokinetics, and risk. Many implementations of OCH PFAS TK are to validate ‘forward’ exposure estimates, in which assessment is performed by applying exposure factors across chemical concentrations in media [61,66,69,70,73]. Commonly cited dynamic models are used to assess variables such as sex [25,77], pregnancy [75], and half-lives among different PFAS [19]. 

**Figure 6:**
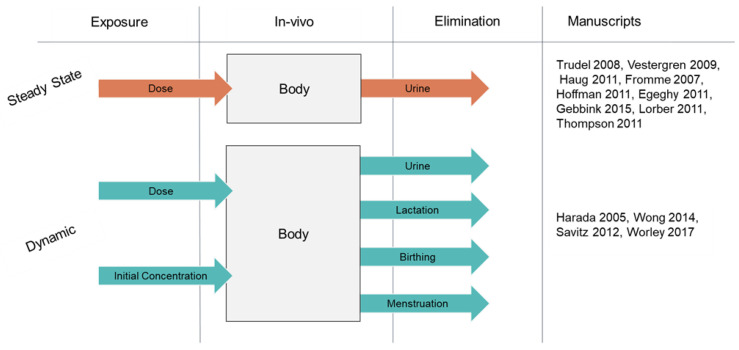
Commonly presented characteristics of OCH TK PFAS models [19,25,61,65,66,67,69,70,72,73,75,77,78].

Derived from the manuscripts in Table 3, Figure 6 provides a visual representation of a generic OCH model The simple, steady state one compartment model—the simplest possible model for toxicokinetics—was the most common. As this model type was often used to evaluate exposure estimates, it is an appropriate approximation for long-term general population scenarios. Time-sensitive events are presented in the dynamic model; as well as a ‘time equal to zero’ initial concentration. Worley et al. [19], Wong et al. [77], and Haug et al. [70] all assert the need for unique modeling scenarios for women as toxicokinetic models differ based on sex. 

The one compartment models presented in the manuscripts listed in Figure 6 are described by Equation (1). In Wong et al. [77], the elimination rate constant in Equation (1) was expanded to include a loss term for menstruation in women, *G_mbl_/V_D_*_,_ where *G_mbl_* is rate of blood serum loss by menstruation and calculated at 6.1 mL/kg-bw/year for women age 12.5 to 50 [77]. The model describes PFOS kinetics for which the authors cite the value of Thompson et al. [67] for *V_D_* of PFOS (230 mL/kg-bw). Thus, units of *G_mbl_/V_D_* (year^−1^) are the same as the elimination rate constant, *k_e_* (year^−1^).

## 4. Discussion

We applied an evidence-mapping approach to characterize the use of TK models of PFAS, with emphasis on simple one compartment models. In our approach to evidence-mapping we applied methods to contextualize the papers retrieved from the scheme in Figure 1; namely, a co-citation network [38,40] based on the 2670 references of our corpus of 92 papers, and a ‘historiograph’ [46] depicting the temporal relationship in citations among the OCH publications. Co-citation analysis and the resulting network provides a mechanism to retrieve relevant papers missed. With this approach we suggest that evidence mapping is more forgiving or tolerant of missed papers compared to systematic review where hypothesis testing of an intervention or exposure scenario is at stake [32,96].

### 4.1. Search Effectiveness

**Figure 7:**
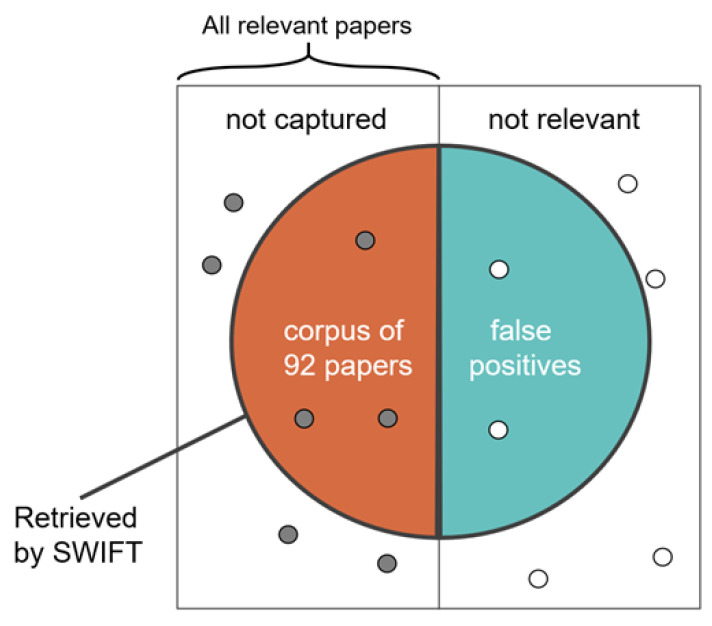
Precision and recall in scoping assessment strategy. Adapted from https://commons.wikimedia.org/wiki/File:Precisionrecall.svg (accessed on 15 December 2022).

As the bibliometric analysis was largely restricted to the corpus of 92 papers, inclusion criteria of PFAS TK models within the collected body impacted the results. The WOS searches and SWIFT review identified a set of potentially relevant papers, shown as the circle in Figure 7. The manual extraction removed false positives, resulting in our corpus of 92 articles. It is possible that utilizations of TK models for PFAS were either not captured in the WOS searches or were incorrectly removed in the SWIFT review tool process. As such, the corpus of PFAS TK papers is presented not as an all-inclusive list of publications within the research focus, but as a strong representative sample from which conclusions and trends are drawn. 

In addition, some models leveraged for TK of PFAS may have originated from other sources not targeted in the search strategy used here. For example, the ‘Ritter Model’ [97] is cited by works in the corpus [77,86], but was developed for polychlorinated biphenyls, and not PFAS. As such, global models developed for other chemicals may not be captured by the search string used in this study. In addition, nomenclature is evolving; in 2004, Butenhoff et al. [55] referred to PFOA by its inorganic precursor, ammonium perfluorooctanoate (APFO) exclusively in the abstract. It is unclear how many documents were missed in this search due to changes in naming conventions, as PFAS were once more commonly known as perfluorochemicals (PFCs). Furthermore, citation of manuscripts does not indicate a repeat in model structure. The links drawn between manuscripts are citations. Some manuscripts discuss a variety of different model structures [37,64], adding to the complexity of drawing conclusions from citations about model selection decisions and influence. A possible application of the co-citation network in *Bibliometrix* is identification of manuscripts that were missed in the initial search, as 2 of the 14 papers identified outside of the corpus implemented TK models. This ‘second screen’ approach may enhance repositories of papers in future searches. 

### 4.2. One Compartment Models: Half-Lives and PFAS as a Class

De Silva et al. [31] found that elimination half-lives are only reliably estimated for 4 PFAS: PFOS, PFOA, PFNA, and PFHxS, which were the PFAS found most often in this study. Although other PFAS were identified during this scoping assessment, the finding in De Silva et al. [31] corroborates that focus has largely centered on 4 long-chain PFAS. In addition, De Silva et al. [31] asserted that exposure assessments oftentimes rely on simple steady state one compartment TK models. This claim is supported by the findings of this scoping assessment.

**Figure 8:**
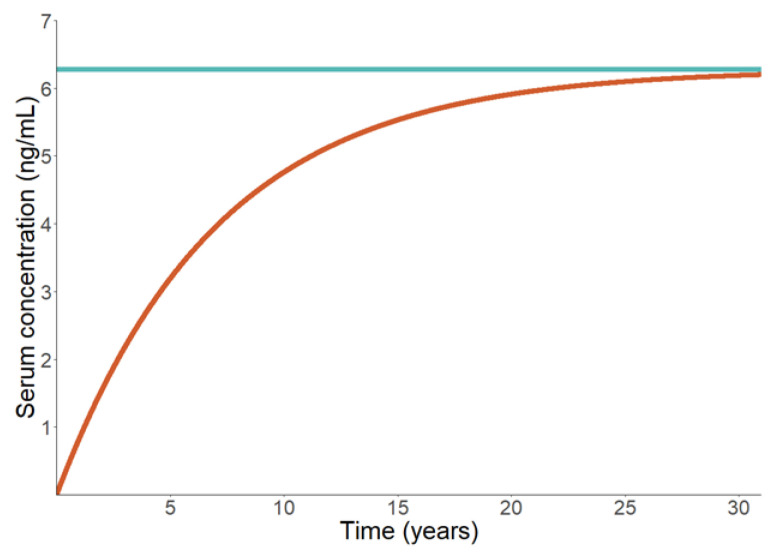
Dynamic (red line) versus steady state (green line) OCH model for PFOS.

Adapted from East et al. [36], Figure 8 illustrates a comparison between a steady-state and dynamic kinetics for a one-compartment PFOS model. The volume of distribution and elimination rates, 230 mL/kg, and 0.00039 1/day were obtained from Andersen et al. [98] and Olsen et al. [56] and used by both Lorber and Egeghy [69] and Egeghy and Lorber [73]. The time-dependent difference between the dynamic model (orange) and the steady state (blue) suggests the impact of going with a steady-state approach when a time-dependent approach is more appropriate. Thus, for example, a steady-state solution may not be effective for modeling children, given their physiological changes over time. Additionally, time-sensitive events, such as occupational exposure, menstruation, and birth are not captured by the simple steady state equation. However, implementation of the OCH steady state TK model is often used as a validation step for exposure estimates, as daily intake estimates are compared against background serum concentrations (Table 3). 

However, modeling approaches and subsequent risk assessment may vary across PFAS given variation in half-lives. A short-chain PFAS, PFBS, has a published half-life as low as one month [20]. In addition, biotransformation is more common among short-chain PFAS, meaning the modeling implications of data-rich, long chain PFAS may not apply to short-chain scenarios [99,100,101,102]. 

### 4.3. Emerging TK Models and Methods

In citations per year (Table S3), Gomis et al. [99] (22), Trudel et al. [61] (19), and Vestergren and Cousins [66] (17) are the most commonly cited papers in the corpus. Trudel et al. [61] apply a one compartment model to estimate consumer exposure while Vestergren and Cousins [66] apply a one compartment model to calculate daily intakes from serum levels. Vestergren and Cousins are both collaborators on the Gomis et al. study [99] which uses a dynamic one compartment model to compare toxic potency in PFAS. Despite its versatility and simplicity, a one-compartment TK model describes first-order kinetics and does not consider saturable processes such as protein binding. Andersen et al. [30] is noteworthy for being first to test the hypothesis that saturable resorption capacity in the kidney could account for the half-life properties of PFOA across species and genders. Andersen et al. [30] employs a two-compartment (central and tissue) structure with a third filtrate compartment to describe renal resorption. Because the model describes a physiological process, saturable renal resorption, we categorized it as a PBTK model; however, it is unlike most PBTK models in that it lumps all non-renal tissue into a single tissue compartment. 

This scoping identified several “full” PBTK structures [63,103,104,105,106,107,108,109] which incorporate saturable renal resorption based on Andersen et al. [30]. To facilitate the QA and evaluation for several of these PBTK models, including those of Fabrega et al. [110], Kim et al. [103,104], and Loccisano et al. [105], all of which were identified in this review, Bernstein et al. [111] developed a template model structure capable of representing each individual model. These include PBTK models of the disposition of PFOA, PFOS, PFHxS, PFNA, and PFDA (perfluorodecanoic acid), in rats and humans. Of the PBTK models captured in this scoping, that of Cheng and Ng [109] is unique in applying a diffusion-limited structure parameterized with transport and protein binding processes based on in vitro data. However, more parsimonious model structures tend be used in regulatory contexts. In its proposed approaches to set a Maximum Contaminant Level Goal (MCLG) for PFOS and PFOA, the EPA Office of Water applied the two-compartment mother-child model of Verner et al. [112] for human dosimetry and applied the model of Wambaugh et al. [113], adapted from Andersen et al. [30], to interpret animal data [112,113,114].

## 5. Conclusions

This scoping assessment identified influential models, generic one compartment model structures, and the breadth of existing publications which apply TK models for PFAS. In addition, a collection of 13 papers which have informed the community of research were identified. Models were classified as one-compartment, two-compartment, PBTK, human or animal, and steady state or dynamic for all 92 papers in the corpus. The complete corpus of papers is available in the supplemental information. 

Characterization of outside influence on model selection for each study is nearly impossible. However, dynamics of TK models for PFAS have been evolving since modeling began. As such, conventional models have been frequently sourced from several authors for toxicokinetics. As modeling challenges increase in complexity, including accounting for PFAS-specific toxicokinetics (long-chain and short-chain), and variations in human populations (e.g., age, sex, menstruation, birthing, breast-feeding), a commensurate effort is required in model selection and implementation. 

Within the corpus, one compartment models are the most common, and feature heavily among the most referenced articles. PFOA and PFOS feature most frequently in TK models. However, calls and efforts to transition to more complex models are growing. This scoping assessment has identified influential papers and has detected trends in the literature which arc towards more granular TK modeling approaches.

## Figures and Tables

**Table 1:** Summary of corpus of extracted texts.

Description	Results
Range of years published	2005–2020
Unique Sources (Journals, Books, etc)	31
Documents	92
Average years from publication	7.45
Average citations per study	41.02
Average citations per year per document	4.406
Total number of references	2670
Unique Authors	358
Authors per study	3.89

**Table 2:** Louvain betweenness of nodes in co-citation network.

	Node	DOI	Between-Ness	GCS	TK Model	Class	Objective
	Olsen GW, 2007 [56]	10.1289/ehp.10009	19.3	760	Yes	Measurement	Estimated half-life of PFOS, PFHS, and PFOA in fluorochemical production workers
	Lau C, 2007 [58]	10.1093/toxsci/kfm128	11.9	2407	No	Review	PFAS concentrations in environment, wildlife and humans. Toxicology and mode of action
	Andersen ME, 2006 [30]	10.1016/j.tox.2006.08.004	11.0	193	Yes	Dynamic PBTK Animal	Developed a PBPK model using renal resoprtion
	Trudel D, 2008 [61]	10.1111/j.1539-6924.2008.01017.x	5.9	472	Yes	Steady State OCH	Estimated Daily intakes of PFOA and PFOS
	Calafat AM, 2007 [50]	10.1289/ehp.10598	5.1	1012	No	Measurement	Shared NHANES PFAS summary statistics
	Butenhoff JL, 2004 [55]	10.1016/j.tox.2003.11.005	3.9	220	No	Measurement	Gestational Rat observations for PFOA
	Emmett EA, 2006 [54]	10.1097/01.jom.0000232486.07658.74	3.6	377	No	Measurement	Determined serum PFOA levels of population living near production facility
	Kudo N, 2002 [28]	10.1016/S0009-2797(02)00006-6	3.5	267	Yes	Dynamic TC Animal	Evaluated role of sex hormones in renal clearance of PFOA in rats
	Han X, 2003 [57]	10.1021/tx034005w	2.9	392	No	Measurement	Examined binding of PFOA to serum albumin in rats and humans
	Bartell SM, 2010 [60]	10.1289/ehp.0901252	2.8	340	Yes	Dynamic OCH	Detemined decline of PFOA in serum samples after filtration intervention in water district
	Karrman A, 2007 [49]	10.1289/ehp.9491	2.7	520	No	Measurement	Compared occurance of PFAS in breastmilk and primiparous women serum
	Ehresman DJ, 2007 [51]	10.1016/j.envres.2006.06.008	2.5	310	No	Measurement	Evaluated PFAS concentration across human blood-based matrices (blood, plasma, serum)
	Prevedouros K, 2006 [4]	10.1021/es0512475	2.5	2374	No	Review	Reviewed fate and transport of PFAS in the environment
	Fromme H, 2009 [59]	10.1016/j.ijheh.2008.04.007	2.5	228	No	Review	Reviewed enviromental and biomonitoring data for PFOS, PFOA and precursors
	Seacat AM, 2002 [48]	10.1093/toxsci/68.1.249	2.4	618	No	Measurement	Identified lowest measurable responses for PFOS in humans using monkeys
	Lou IC, 2009 [64]	10.1093/toxsci/kfn234	2.0	97	Yes	Dynamic OCH, TCH, PBTK	Characterize pharmacokinetics of PFOA in mice to estimate exposure
	Tan YM, 2008 [62]	10.1016/j.toxlet.2007.12.007	1.9	70	Yes	PBTK Animal	Evaluated determinants of disposition of PFOA and PFOS in rats, monkeys
	Loccisano AE, 2011 [63]	10.1016/j.yrtph.2010.12.004	1.7	94	Yes	PBTK Animal/Human	Predicts/evaluates PFOA/PFOS pharmacokinetics for monkeys and humans
	Vandenheuvel JP, 1991 [53]	10.1002/jbt.2570060202	1.5	278	No	Measurement	Exploration of sex differences in elimination of PFOA in rats
	Weaver YM, 2010 [52]	10.1093/toxsci/kfp275	1.4	126	No	Measurement	Evaluated PFAS of differing chain length as substrates of renal transporters

Note: blue = true positives, within the corpus; yellow = true negatives, outside corpus, red = false negatives, outside corpus but leveraged TK model for PFAS (search missed). GCS= Global citations. Counts of citations from Google Scholar, 9 December 2022.

**Table 3:** One Compartment Human Models with over 50 Global Citations.

Document	DOI	GCS	LCS	Model Structure	Model Purpose
Trudel D, 2008 [61]	10.1111/j.1539-6924.2008.01017.x	286	15	Steady State	Validation of exposure estimates
Vestergren R, 2009 [66]	10.1021/es900228k	242	9	Steady State	Validation of exposure estimates
Haug LS, 2011 [70]	10.1016/j.envint.2011.01.011	203	7	Steady State	Validation of exposure estimates
Harada K, 2005, [25]	10.1016/j.envres.2004.12.003	171	10	Dynamic	Evaluation of sex-based differences in elimination
Fromme H, 2007 [65]	10.1021/es071244n	163	10	Steady State	Validation of exposure estimates
Wong F, 2014 [77]	10.1021/es500796y	90	6	Dynamic	Evaluation of sex-based differences in elimination
Hoffman K, 2011 [72]	10.1289/ehp.1002503	90	0	Steady State	Estimate relative contributions of contaminated drinking water to serum concentation
Savitz DA, 2012 [75]	10.1097/EDE.0b013e31824cb93b	82	3	Dynamic	Assess association between exposure and pregnancy outcomes
Egeghy PP, 2011 [73]	10.1038/jes.2009.73	79	0	Steady State	Validation of exposure estimates
Worley RR, 2017 [19]	10.1016/j.envint.2017.06.007	68	2	Dynamic	Determination of half-lifes to characterize exposure
Gebbink WA, 2015 [78]	10.1016/j.envint.2014.10.013	66	5	Steady State	Identification of relative source contributions and precursors
Lorber M, 2011 [69]	10.1021/es103718h	60	7	Steady State	Validation of exposure estimates
Thompson J, 2010 [67]	10.1016/j.envint.2010.02.008	56	14	Steady State	Characterization of exposure from serum concentrations

## Data Availability

Not applicable.

## Supplementary Materials

The following supporting information can be downloaded at: https://www.mdpi.com/article/10.3390/toxics11020163/s1, Figure S1: Search String; Figure S2: Ranking Performance; Table S1: Systematic Map; Table S2: Data; Table S3: Corpus Citations; Table S4: Co-Citation Top 20.
